# Immunosuppressants Tacrolimus and Sirolimus revert the cardiac antifibrotic properties of p38-MAPK inhibition in 3D-multicellular human iPSC-heart organoids

**DOI:** 10.3389/fcell.2022.1001453

**Published:** 2022-11-11

**Authors:** Yu Tian, Yuta Tsujisaka, Vanessa Y. Li, Kanae Tani, Antonio Lucena-Cacace, Yoshinori Yoshida

**Affiliations:** ^1^ Center for iPS Cell Research and Application, Kyoto University, Kyoto, Japan; ^2^ Graduate School of Medicine, Kyoto University, Kyoto, Japan; ^3^ Department of Cardiovascular Medicine, Graduate School of Medicine, Kyoto University, Kyoto, Japan; ^4^ Wellesley College, Wellesley, MA, United States

**Keywords:** heart organoid, sirolimus, tacrolimus, p38, cardiac fibroblasts, fibrosis, SB202190

## Abstract

Cardiac reactive fibrosis is a fibroblast-derived maladaptive process to tissue injury that exacerbates an uncontrolled deposition of large amounts of extracellular matrix (ECM) around cardiomyocytes and vascular cells, being recognized as a pathological entity of morbidity and mortality. Cardiac fibrosis is partially controlled through the sustained activation of TGF-β1 through IL-11 in fibroblasts. Yet, preclinical studies on fibrosis treatment require human physiological approaches due to the multicellular crosstalk between cells and tissues in the heart. Here, we leveraged an iPSC-derived multi-lineage human heart organoid (hHO) platform composed of different cardiac cell types to set the basis of a preclinical model for evaluating drug cardiotoxicity and assessing cardiac fibrosis phenotypes. We found that the inhibition of the p38-MAPK pathway significantly reduces COL1A1 depositions. Yet, concomitant treatment with organ-rejection immunosuppressant drugs Tacrolimus or Sirolimus reverts this effect, opening new questions on the clinical considerations of combined therapies in reducing fibrosis after organ transplantation.

## Introduction

Cardiac fibroblasts (CFs) constitute a significant cell population conforming to the myocardium (Moore-Morris et al., 2016). During cardiogenesis, CFs play crucial functions in stimulating cardiac myocyte proliferation and providing the extracellular matrix (ECM) scaffold that serves as structural support where various cellular components of the heart are organized (Ieda et al., 2009). The quantity and quality of the ECM depositions are vital factors defining normal developmental cardiac behavior and indicative predictors of cardiac functionality in heart failure following fibrosis (Ieda et al., 2009). Heart failure leads to cardiac remodeling to compensate for the cellular loss of affected areas of the myocardium (Roger et al., 2012). Cardiac remodeling requires the activation of several cellular processes involving cardiac myocyte hypertrophy, infiltration of immune cells, and cardiac fibrosis, where CFs are the key players. During heart failure, two distinctive processes involving CFs-driven remodeling trigger: Replacement fibrosis and reactive fibrosis. Reactive fibrosis is considered a pathological response of a maladaptive process where CFs deposit large amounts of ECM around cardiomyocytes and vascular cells, increasing tissue rigidity, and is recognized as a pathological entity in morbidity and mortality (Jellis et al., 2010). However, to ensure the structural integrity of the affected heart chamber, replacement fibrosis plays an essential role by scarring, which ultimately helps the heart compensate for functionality upon a massive loss of cardiomyocytes lacking regenerative capacity (Graham-Brown et al., 2017).

CFs arise from multiple origins during development, which makes them a heterogenetic complex cell lineage (Rinn et al., 2006; Ali et al., 2014). Despite a small subset of CFs being known to derive from the endocardium and the cardiac neural crest, most CFs derive from the epicardium, a protective epithelial layer covering the four cardiac chambers (Mikawa and Gourdie, 1996; Gittenberger-de Groot et al., 1998; Cai et al., 2008; Smith et al., 2011; Cao and Poss, 2018) entirely. The epicardium has emerged as an essential player in the cardiac repair and regeneration (Cao and Poss, 2018), being a critical cell source of progenitors of different cardiac lineages, including CFs (Mikawa and Fischman, 1992; Mikawa and Gourdie, 1996; Gittenberger-de Groot et al., 1998; Cai et al., 2008; Zhou et al., 2008; Smith et al., 2011). Taken altogether, the sole usage of fibroblast cell lines to study cardiac fibrosis could foster a bias of an oversimplified model that excludes fibroblast heterogeneity and the multicellular nature of the different cells mediating signaling promoting fibroblast-derived ECM depositions in developmental, pathological, and regenerative responses in the human heart.

Fibrotic-derived ECM is a non-cellular 3D complex composed of elastin, fibronectin, proteoglycans/glycosaminoglycans, collagens, and other glycoproteins (Frantz et al., 2010). Collagen type I α1 (COL1A1) is the major component of ECM depositions found in most embryonic and connective tissues, including reactive fibrosis (Gimenez et al., 2017; Heras-Bautista et al., 2019; Fu et al., 2020; Chen et al., 2021). TGF-β1 is one of the most potent pathological activators of COL1A1 fostering ECM depositions (Khalil et al., 2017), and this effect is finely controlled through interleukin-11 (IL-11) (Schafer et al., 2017), which can be either produced cell-autonomously or as a result of a secretory phenotype of nearby cells in the human heart as a multicellular organ. We found that activation of the RAS pathway in cardiac fibroblast is intrinsically associated with collagen type I production, in particular through PI3K/AKT/mTORC1, Calcium signaling, and p38-MAPK pathways.

Here, we have used an iPSC-derived multi-lineage human heart organoid (hHO) platform (Lewis-Israeli et al., 2021) to assess the effectiveness of the immunosuppressants Tacrolimus (Calcineurin inhibitor) and Sirolimus (mTORC1 inhibitor) as well as SB202190, a selective p38-MAPK inhibitor (p38 2i α/β isoforms) for the treatment of fibrosis in hHO stimulated with TGF-β1.

Finally, we identified that inhibition of p38-MAPK signaling is able to suppress COL1A1 expression in hHOs upon pathologic stimulation with TGF-β1, yet the concomitant therapy of SB202190 with either Tacrolimus or Sirolimus reverts this phenotype, nullifying its anti-fibrotic effects.

## Materials and methods

### Cell culture and human heart organoid induction

Human Ventricular Cardiac Fibroblasts (NHCF-V) were purchased from Lonza (CC-2904) and cultured under manufacturer indications. Feeder-free hiPSCs (1390C1) were maintained in StemFit^®^ AK02N medium (Reprocell) on iMatrix-511 (nippi) coated plates. Human heart organoids were differentiated from PSCs by using a published method. Briefly, iPSCs were dissociated with Accumax and resuspended in Essential 8 Flex medium (Gibco) containing 10 μM ROCK inhibitor Y-27632. To generate EBs, 10,000 cells were seeded at a final volume of 100 μl per well in round bottom low-attachment HEMA-coated 96-well plates on day -2. Fresh Essential 8 Flex medium was added the next day. On day 0, Essential 8 Flex medium was removed, and differentiation was performed in RPMI 1640/B-27, minus insulin (Gibco) containing CHIR99021 (4 μM), BMP4 (1.25 ng/ml) and ActivinA (1 ng/ml). On day 1, the medium was replaced with fresh RPMI 1640/B-27, minus insulin. On day 2, the medium was changed to RPMI 1640/B-27, minus insulin containing Wnt-C59 (2 μM). On day 4, the medium was replaced with fresh RPMI 1640/B-27, minus insulin. On day 6, the medium was replaced with fresh RPMI 1640/B-27 (Gibco). On day 7, organoids were treated with 2 μM CHIR99021 in RPMI 1640/B-27 for 1 h. From day 7 onwards, the medium was changed every other day until day 15.

### Cytotoxic assay in cardiac fibroblasts

SB202190, Tacrolimus, and Sirolimus were freshly and individually prepared in DMSO for each individual experiment. Cardiac fibroblasts were seeded individually in 96-well plates (5,000 cells per well). The treatment effectiveness was assayed after applying reducing concentrations in a 1:2 fixed ratio to cycling cells in the exponential phase. All treatments were assessed within reducing doses of SB202190, Tacrolimus, or Sirolimus in monotherapy or combination in fixed proportions a day after the seeding. The proliferation viability was determined by crystal violet after 96 h. Cytotoxicity profiles were measured by absorbance at 595 nm using a microplate reader; then, IC50 values were estimated using GraphPad Prism 7 software.

### Cardiotoxicity assays in iPSC-derived human heart organoids

SB202190, Tacrolimus, and Sirolimus were prepared alone or in combinations mixed in DMSO for each experiment. hHOs were differentiated and seeded separately in low-binding 96-w plates. The effectiveness of the treatment was measured after the usage of decreasing concentrations in a 1:2 ratio to day 15 of developing heart organoids. All treatments were applied by reducing dosages of SB202190, Tacrolimus, or Sirolimus in monotherapy or combination. The proliferation capacity as a functional assay was determined using an MTT assay after 96 h (4 days). Cytotoxicity profiling was drafted by obtaining absorbance metrics at 595 nm using a particular microplate reader; then, IC50 values were calculated using GraphPad Prism 7.

For organoid beating evaluation, we measured the beating count at 10 s intervals and then compared it to the beating count of the untreated organoid for each case in biological triplicates.

### Immunocytochemistry (ICC)

Cells were fixed by 4% PFA treatment for 15 min and stored in PBS at 4°C. Blocking was performed for 30–45 min in a blocking buffer containing 1% BSA, 0.5% Triton X-100 and 0.1 M glycine in PBS. After washing three times with PBS, primary antibody anti-Collagen I (1:200, ab34710, Abcam) was added to the blocking buffer (w/o glycine) and incubated overnight at 4°C. The next day, cells were washed three times with PBS, and secondary antibody donkey anti-rabbit Alexa Fluro 594 (1:1000, A21207, Invitrogen) was added in 1% BSA-PBS for 2 h at RT. After washing three times with PBS, 1:10,000 Hoechst 33342 was added for nuclear counterstaining. Images were taken with Keyence.

Immunofluorescence staining of hHOs was conducted as previously described. hHOs were fixed by 4% PFA solution for 45–60 min at room temperature and incubated in blocking/permeabilization solution containing 10% normal donkey serum (Abcam), 0.5% Triton X-100 (nacalai tesque) and 0.5% BSA (nacalai tesque) at 4°C overnight. Subsequently, hHOs were stained with primary and secondary antibodies in PBS containing 1% normal donkey serum, 0.5% Triton X-100 and 0.5% BSA, respectively. Nuclei were stained with Hoechst 33342 (1:1000) before mounting. A confocal laser scanning microscope (Olympus FV3000) was used for fluorescent observation. Primary antibodies used are listed as follows: anti-Vimentin (1:200, ab11256, Abcam); anti-Collagen I (1:200, ab34710, Abcam); anti-WT1 (1:200, ab89901, Abcam); anti-ZO-1 (1:200, 33-9100, Invitrogen); anti-CD31/PECAM1 (1:50, ab28364, Abcam); anti-Cardiac Troponin T (1:200, ab8295, Abcam); anti-NFAT2 (1:200, ab25916, Abcam); anti-CD90/THY1 (1:200, ab133350, Abcam). Secondary antibodies used are listed as follows: donkey anti-mouse Alexa-Fluor 488 (1:200, A21202, Invitrogen); donkey anti-rabbit Alexa-Fluor 594 (1:200, A21207, Invitrogen); donkey anti-goat Alexa-Fluor 488 (1:200, A11055, Invitrogen); donkey anti-goat Alexa-Fluor 647 (1:200, A21447, Invitrogen).

### Immunoblotting

Cells were lysed in M-PER Mammalian Protein Extraction Reagent (78501, Thermo Scientific) buffer, and hHOs were lysed in T-PER Tissue Protein Extraction Reagent (78510, Thermo Scientific). The amount of protein was determined by Bradford assay using BSA as a standard. The primary antibodies were purchased from commercial sources as follows: anti-phospho-S6 Ribosomal Protein (1:1000, 5364, CST); anti-phospho-4E-BP1 (1:1000, 2855, CST); anti-4E-BP1 (1:1000, 9644, CST); anti-phospho-p38 (1:1000, 4631, CST); anti-p38 (1:1000, 9212, CST); anti-phospho-AKT (1:1000, 9271, CST); anti-Collagen I (1:1000, ab34710, abcam); anti-IL-11 (1:1000, ab76589, abcam); anti-β-actin (1:1000, A5441, Sigma). The secondary antibodies used are as follows: goat anti-rabbit IgG (HRP) (1:5000, sc-2054, Santa cruz), and anti-mouse IgG (HRP) (1:5000, 7076, CST).

### Image acquisition, processing, and analysis

Microscopy images were taken by BZ-X710 and processed by BZ-X Analyzer (Keyence); pseudo-coloring was used as indicated in figure legends. A representative section was chosen, cropped, and magnified for phase contrast and fluorescence pictures. Western blot data were recorded by ImageQuant 800 (Cytiva). General image analysis was performed using Microsoft power point as well as ImageJ v1.52.

### Bioinformatics

Retrospective bioinformatical analysis performed in this study was conducted using R2: Genomics analysis and visualization platform (http://r2.amc.nl/). The publicly available datasets used in this study for the retrospective analysis are GSE133452, GSE132146, and GSE97458.

### Quantitative RT-PCR

According to the manufacturer’s manual, total RNA was extracted using QIAzol^®^ Lysis Reagent (Qiagen). 1 μg RNA was reverse transcribed into cDNA using the ReverTra Ace system (Toyobo BIOTECH). Quantitative RT-PCR (qPCR) was done with Next SYBR^®^ qPCR Mix (Thunderbird) using the StepOnePlus Real-Time PCR system. GAPDH was used as an endogenous housekeeping control. Primer sequences are listed in Supplementary Table S1.

## Results

### Retrospective analysis of mouse and human cardiac fibroblast transcriptomics reveals different branches of the RAS signaling pathway implicated in Col1a1 activation

**FIGURE 1:**
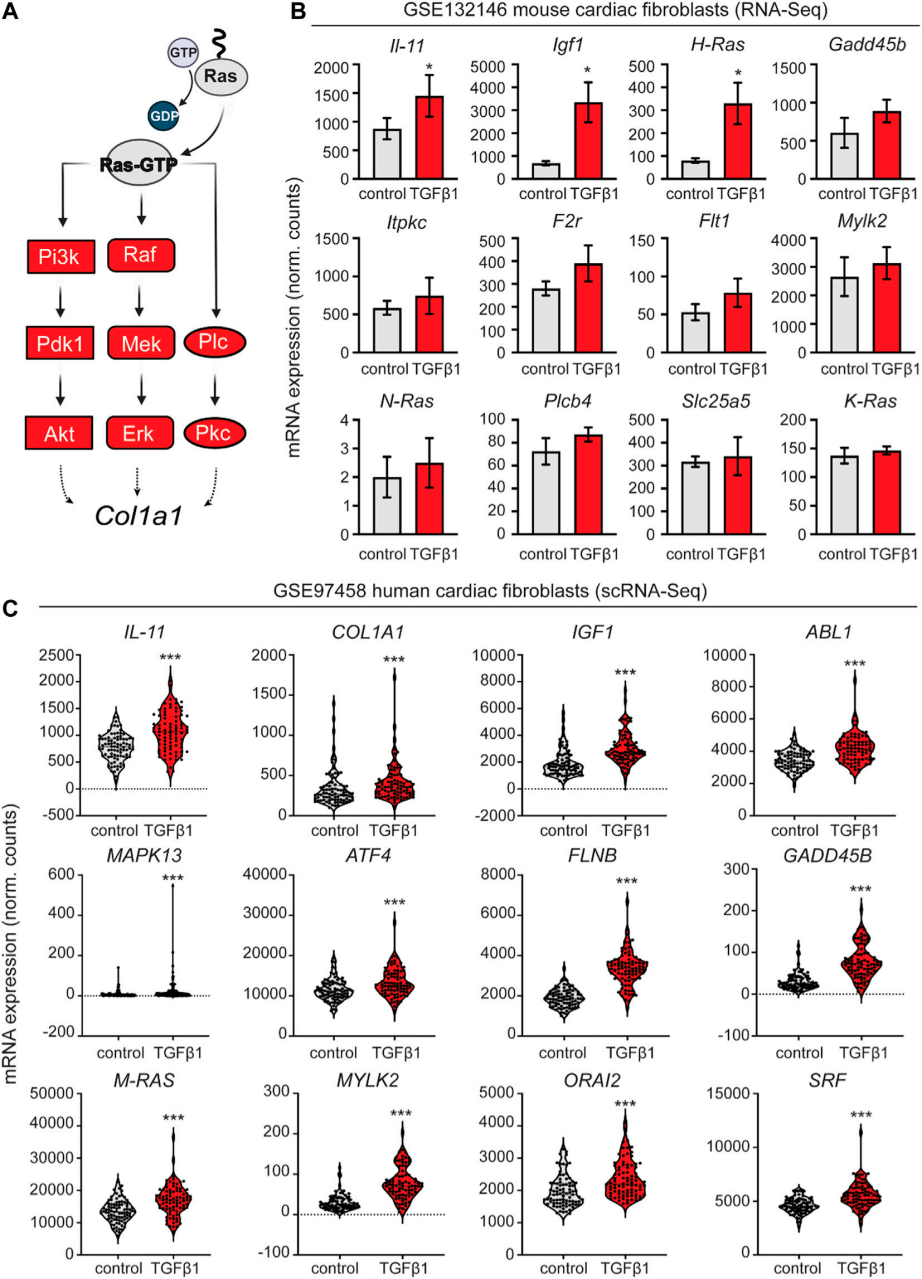
Retrospective analysis of transcriptomic data from mouse and human cardiac fibroblasts activated with TGF-β1. **(A)** schematic representation of the activated nodes of the Ras signaling pathway. **(B)** Expression analysis of the mouse cardiac fibroblasts dataset GSE132146 showing transcriptional levels of pro-fibrotic and RAS pathway-related genes (*, *p* < 0.05; **, *p* < 0.01; ***, *p* < 0.001 compared to control). **(C)** Violin plots showing pro-fibrotic and RAS pathway related genes derived from a retroactive analysis of GSE97458 generated from single-cell RNA-seq of serum-free cultured human cardiac fibroblasts activated with TGF-β1 (*, *p* < 0.05; **, *p* < 0.01; ***, *p* < 0.001 compared to control).

We aimed to identify developmental transcriptomic inroads for establishing CFs cell lineage and ECM deposition of fetal fibroblasts in a pathological context of TGFβ-1 driven fibrosis. Classically, signal transduction in the RAS pathway has been associated with reactive fibrosis responses in disease through the proven effects of Farnesythiosalicylic acid (FTS) acting as a RAS antagonist, decreasing inflammation and fibrosis in animal models (Reif et al., 1999; Katzav et al., 2001; Clarke et al., 2003; Reif et al., 2004; Kafri et al., 2005; Nevo et al., 2011; Rokni et al., 2022). First, to confirm the implications of the RAS pathway and its branches in fibrotic cardiac disease (Figure 1A), we retrospectively analyzed publicly available RNA-Seq data with relevant transcriptomic profiling on mouse cardiac fibroblasts activated upon TGF-β1 treatment [GSE132146 (Ruiz-Villalba et al., 2020)]. We confirmed significant upregulation of Il-11, Igf1, and H-Ras transcripts (Figure 1B) and increased elevation of several markers identified as essential regulators of the different branches of the RAS signaling pathway.

Next, to contextualize our experiments in a human disease setting, we validated several key markers such as IL-11, IGF1, and MAPK13, among others, through a retroactive analysis of GSE97358 (Figure 1C), a single-cell RNA-seq data of cardiac fibroblasts cultured in serum-free media during 16 h before TGF-β1 treatment (Schafer et al., 2017).

CMs and CFs share a common progenitor early in development, the cardiac mesodermal progenitor cells [KDR^+^, PDGFRα^+^, KIT^+^; PMID (Doyle et al., 2015)]. Inactivation of the p38-MAPK signaling is involved in the progression to the multipotent cardiac progenitor cells when differentiating to the cardiac myocyte lineage, so we explored how the transdifferentiation model implies the reactivation of the p38-MAPK and RAS signaling pathway in the establishment of the fibroblast program, a rapidly-cycling developmental program that is, intrinsically associated to the establishment of the fetal fibrotic program and production of ECM (Vancheri, 2012). The multicellular composition of the heart represents a problem relying on cellular heterogeneity, so understanding bidirectional developmental transitions in molecular trajectories between healthy cells and how these regulate the activation or suppression of pathways initiating fibrosis could represent an interesting and complementary molecular approach. Since cellular transdifferentiation represents a switch in developmental commitment between cell identities, investigating pathway regulatory mechanisms between cells with a common cardiac progenitor (*Isl1* ^+^, *Nkx2.5* ^+^, *Flk-1* ^+^), such as cardiac fibroblasts (CFs) and cardiomyocytes (CMs) could provide a novel opportunity to explore divergences in cellular and molecular mechanisms that may underlie specification of gene expression in health (replacement fibrosis) and disease (reactive fibrosis). To understand the molecular divergences leading to the activation of ECM-related fibrosis between CFs and fibroblast-transdifferentiated lineages into cardiomyocytes, we systematically explored differentially expressed genes and pathways in a single cell expression model (GSE133452) that contains a human pluripotent stem cell (hPSC)-based transdifferentiation model of cardiomyocytes from CFs through the forced expression of *Gata4*, *Mef2c*, and *Tbx5* (GMT) (Stone et al., 2019). First, we confirmed that while CFs expressed lineage-specific genes such as *Col1a1*, *Fap*, *Fn1*, *Pdgfra*, or *Tcf21* (Supplementary Figure S1A top), transdifferentiated cardiomyocytes expressed cardiac myocytes key markers including *Myh6*, *Myl4*, *Myl7*, *Tnni3* and *Tnnt2* (Supplementary Figure S1A bottom). Next, differentially expressed pathways between the two groups confirmed the RAS signaling as one of the primarily differentiated active pathways in CFs, indicating that suppression of this pathway in CMs plays an important developmental role in terms of proliferation, lineage specification, and functional maturation (Supplementary Figure S1B). Activation of the RAS signaling pathway suggests the potential activation of its downstream axes, including the PI3K/Akt/mTOR pathway, Raf/MEK/ERK/MAPK pathway, and PKC/Calcium signaling pathway. Heatmaps of differentially expressed genes in these pathways confirmed the up-regulations of important mediators and effectors for their activation (Supplementary Figure S1C). Of particular interest, we found up-regulated transcripts of Vdac1/2 related to Calcium signaling and Mapk11, 13, and 14, encoding for different isoforms of the MAP-kinase p38 (*β*, *γ* and *α* isoforms, respectively), indicating that both Calcium signaling and p38-MAPK have important roles in the establishment of the fibrosis pathway. RGS proteins have been proposed to have implications in establishing the fibrosis transcriptional program in cardiac fibroblasts upstream p38-MAPK pathway, mainly *via* the *Rgs4* expression (Miao et al., 2016; Carbone et al., 2022). We analyzed RGS gene expression levels and found that most of them are poorly represented in healthy CFs (Supplementary Figure S2A).

Finally, we exploited gene correlations at a single cell level by sorting out cells by *Col1a1* mRNA expression levels. *Col1a1* was mostly CF specific and displayed a significant positive correlation to HRas (*R* = 0.420), Akt1 (*R* = 0.356), Mapk14 (*R* = 0.132) and Vdac2 (*R* = 0.319) (Supplementary Figure S1D), highlighting important RAS downstream effectors as potential druggable pathways for the clinical amelioration of reactive fibrosis upon pathological stimuli.

### TGF-β1 driven COL1A1 expression can be attenuated by SB202190 alone and in combination with immunosuppressors in a primary line of human cardiac fibroblast

**FIGURE 2:**
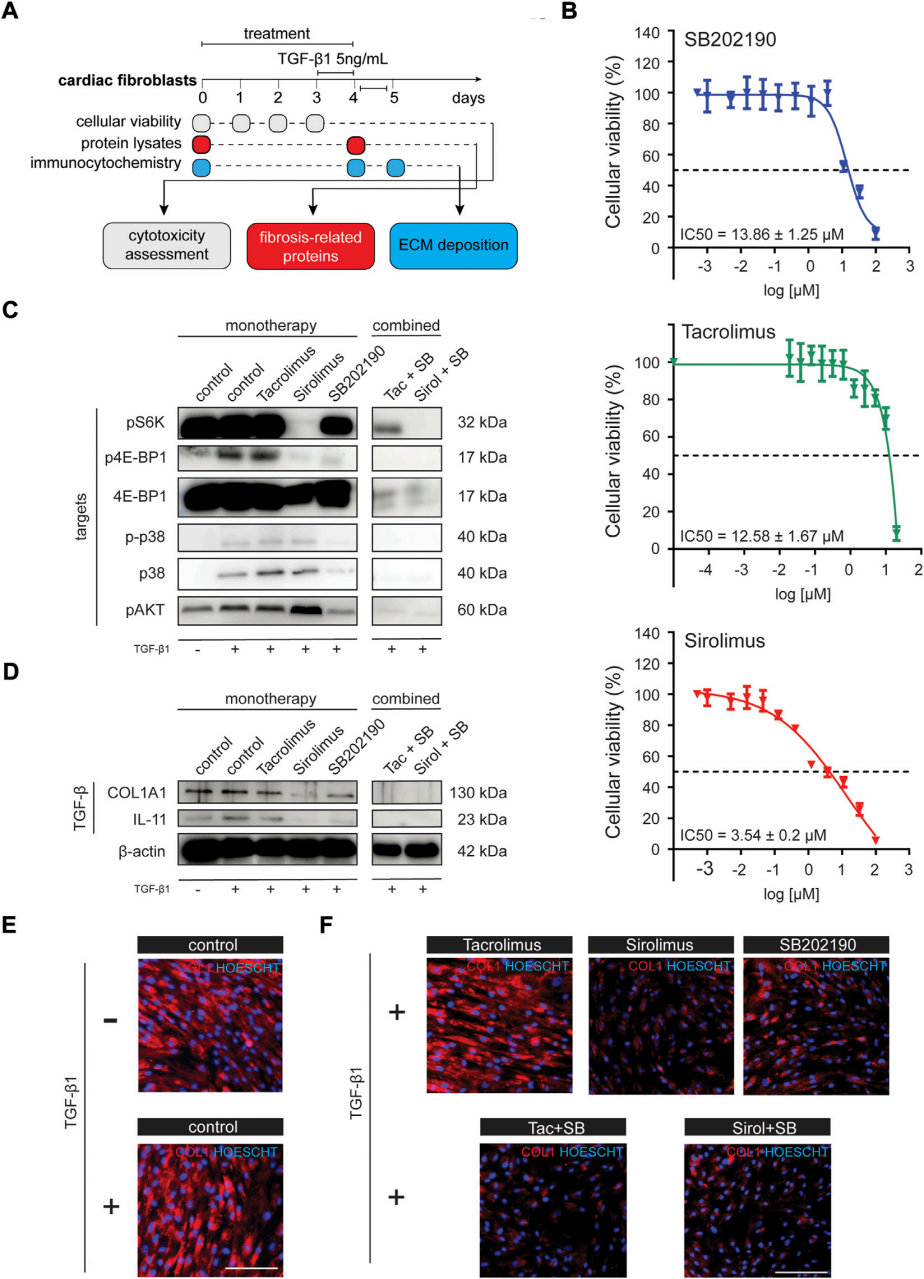
Chemical evaluation of antifibrotic compounds in cardiac fibroblast primary human line activated with TGF-β1. **(A)** Schematic representation of the experimental workflow. **(B)** IC50 assay (96 h) for the evaluation of the cytotoxic profile of SB202190, Tacrolimus and Sirolimus in NHCF-V cell line. **(C)** Western blot analysis in NHCF-V cell line for pathway targets upon different treatments and TGF-β1 stimulation [pS6K, p4E-BP1, 4E-BP1, p-p38, p38, pAkt; β-actin is used as a loading control derived from the same experiments in Figure 3D]. **(D)** Western blot analysis in NHCF-V cell line for evaluation of cardiac fibrosis and TGF-β1 responsive genes [COL1A1, IL-11]. **(E)** Immunocytochemistry analysis of COL1A1 expression in NHCF-V upon 5 ng/ml TGF-β1 stimulation for 24 h. **(F)** Immunocytochemistry analysis of COL1A1 expression in NHCF-V treated with SB202190 (10 μM), Tacrolimus (10 μM) and Sirolimus (200 nM) and 5 ng/ml TGF-β1 stimulation for 24 h [scale bar: 50 μm].

To explore the causal relationship of the RAS signaling activation in the context of cardiac fibrosis and the generation of fibroblast-derived ECM-proteins, we systematically investigate the inhibition of the different downstream axis of RAS in activated fibroblasts upon TGF-β1 stimulation (Figure 2A). To that end, we used a primary cell line of human cardiac fibroblast and explored the inhibition of PI3K/Akt/mTORC1 signaling through rapamycin (Sirolimus), a macrolide used to coat coronary stents to prevent organ rejection after transplantation. The PKC/Calcium signaling was inhibited through FK506 (Tacrolimus; TAC), a calcineurin inhibitor and another common immunosuppressant used as concomitant treatment in patients that received organ transplantation, particularly kidney transplants. TAC can also partially inhibit mTOR signaling by reducing phospho-Akt and interacting (preventing) with the activation of p38. The p38 MAPK/ERK signaling pathway was inhibited using SB202190, a selective inhibitor of p38α/β isoforms (p38 2i). First, we characterized the cytotoxicity profile of these compounds on CF viability. Cytotoxicity assessment showed that CFs had a moderate tolerance to SB202190 and Tacrolimus (TAC), with an IC50 value of 13.86 and 12.58 μM, respectively (Figure 2B). Based on these results, we worked with IC50-suboptimal concentrations, specifically 10 μM for SB202190 and TAC (Figure 2C). Sirolimus was used in 200 nM doses; an effective concentration proved to inhibit the mTORC1 downstream targets p4E-BP1 and pS6K (Figure 2C). We also verified that combined treatments ensured high cellular viability in CFs after 96 h (Supplementary Figures S3A,B). After 3 days of treatment on unstimulated CFs, we treated cells with 5 ng/ml of TGF-β1 in combination with the inhibitors for another 24 h to recapitulate pathological conditions of heart disease and induced a forced expression of ECMs, with a particular focus on COL1A1 as a major component of the Collagen type I.

**FIGURE 3:**
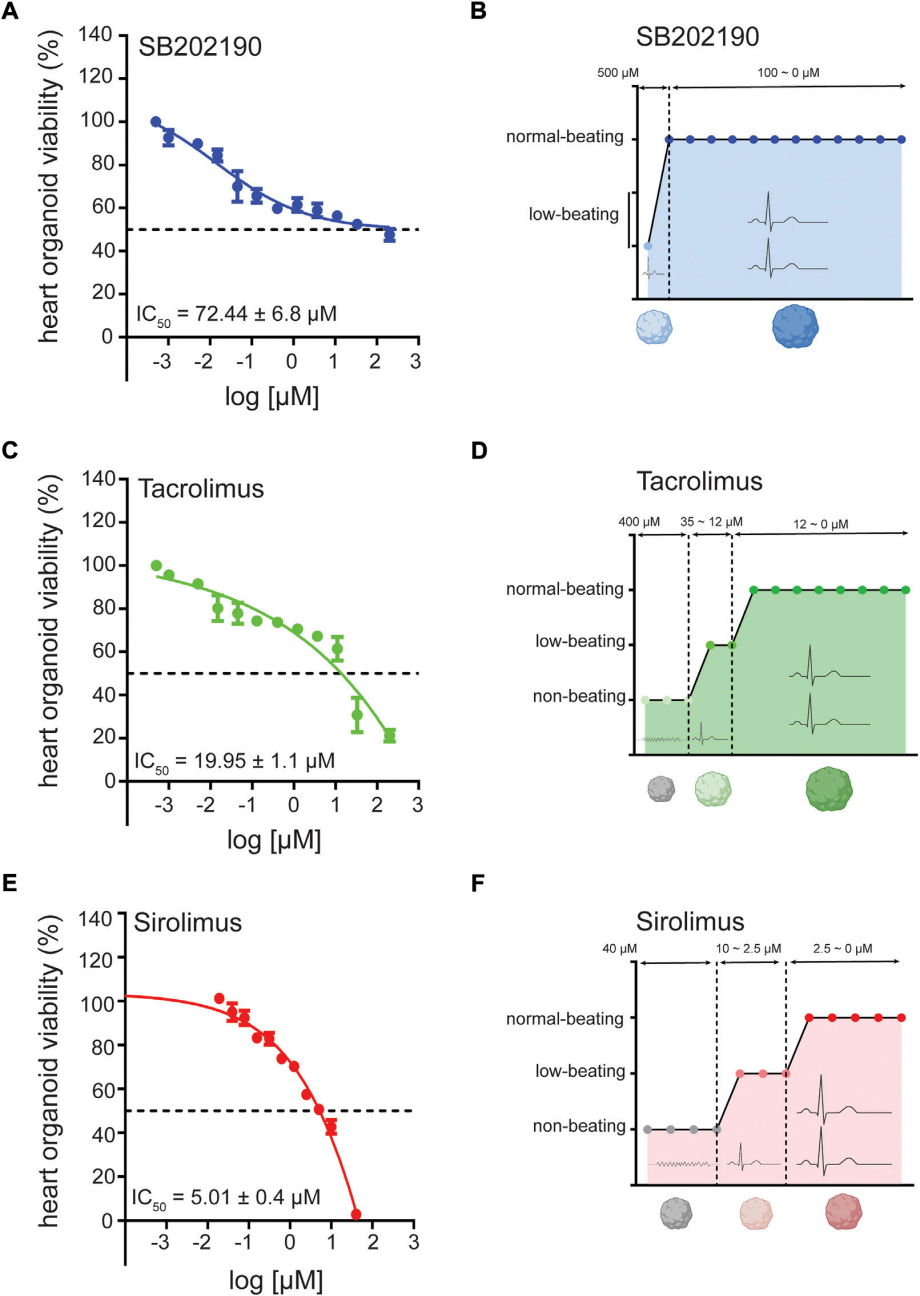
Cardiotoxicity of antifibrotic compounds in human heart organoids (hHOs) for disease modeling. **(A)** MTT assay showing hHO cellular viability and IC50 after 96 h of treatment with SB202190 in decreasing concentrations at fixed 1:2 dilutions from 10 mM. **(B)** compound dose-profiling for electrophysiological beating perturbation in hHO under decreasing concentrations of SB202190 for 96 h. **(C)** MTT assay showing hHO cellular viability and IC50 after 96 h of treatment with Tacrolimus in decreasing concentrations at fixed 1:2 dilutions from 10 mM. **(D)** compound dose-profiling for electrophysiological beating perturbation in hHO under decreasing concentrations of Tacrolimus for 96 h. **(E)** MTT assay showing hHO cellular viability and IC50 after 96 h of treatment with Sirolimus in decreasing concentrations at fixed 1:2 dilutions from 2 mM. **(F)** compound dose-profiling for electrophysiological beating perturbation in hHO under decreasing concentrations of Sirolimus for 96 h.

Although IL-11 increments are increased explicitly in TGF-β1 treated fibroblasts (Figure 2D) as a significant fibrotic trigger, the increments on COL1A1 protein levels are not observable either by western blot or immunostaining (Figures 2D, 3E). To confirm the induction of the fibrotic-like response, we confirmed the up-regulation of pro-fibrotic related genes such as *IL-11*, *VIM,* and *POSTN* (Supplementary Figure S4A) as well as the increments on some genes related to the activation of the RAS pathway such as *IGF1* and *SPHK1* (Supplementary Figure S4B).

Whereas Sirolimus and SB202190 had an independent effect in reducing COL1A1 protein expression, TAC showed no effect in ameliorating the fibrotic phenotype in monotherapy (Figures 2D–F). Indeed, TAC showed to mildly increase COL1A1 levels in the TGF-β1 unstimulated condition, pointing out a pro-fibrotic effect (Supplementary Figure S5A) in health, consistent with a study that explores the induced perturbations of immunosuppressive drugs on the human hearts using human iPSC-derived heart organoids (Sallam et al., 2022). Next, we tested whether the combination of SB202190 with the immunosuppressors could foster the reduction of ECM production since inhibition of one or more downstream axis of RAS could enhance the antifibrotic properties of the treatment. Interestingly, we found that the combined therapy of SB202190 with either TAC or Sirolimus had a profound impact on reducing COL1A1 expression (Figures 2D–F), suggesting that p38-MAPK has an independent role in the activation of reactive fibrosis from Calcium signaling and mTORC1. Importantly, COL1A1 expression correlated with levels of interleukin-11 (IL-11), a key factor mediating fibrotic responses in the human heart (Figure 2D). These results indicate that inhibiting one or more downstream axis of RAS could ameliorate maladaptive responses to the hyperproduction of ECM in the pathological context of cardiac fibrosis.

### 3D organoid cardiotoxicity assays *in vitro* reveal moderate to high tolerance to Tacrolimus and SB202190

Next, to verify the potential efficacy of these compounds in a more physiological setting that recreates pathological disorders, we generated a self-assembling human heart organoid (hHO) from human iPS cell lines. A recently reported model helps to create developmentally relevant settings to study cardiac responses in the health and disease (Lewis-Israeli et al., 2021). Through three-step Wnt signaling modulation, we differentiated the feeder-free human iPS-cell line 1390C1 onto hHOs to obtain 80%–90% of beating organoids on day 6, further increased to 100% by day 10. On day 15, we assessed the multicellular composition and degree of vascularization of the hHO model. Immunocytochemistry (ICC) revealed TNNT2^+^ populations marking beating cardiomyocytes, WT1^+^ cells for human epicardium, CD31^+^ cells for endothelium, NFATC1^+^ for endocardial cells, and VIM^+^ for cardiac fibroblast populations (Supplementary Figure S6), with little changes in cellular composition ratios between differentiation batches. We then characterized the cardio-cytotoxicity profiles of these compounds to different treatment dosages for 96 h *in vitro* through an MTT assay. Generally, we found that hHOs had enhanced cytoprotecting properties against compounds from treated CFs in isolation as a monolayer. This phenotype could be partially explained due to the organoid density as a physical barrier for compound penetration and permeabilization. Also, the tissue environment and the signaling between different cells might confer higher resistance to cell death. IC50 values were higher than those in CFs for each compound, being SB202190 and TAC, the chemicals that showed a moderate to high tolerance for organoid viability, with IC50 of 72.44 and 19.95 μM, respectively (Figures 3A–D). Sirolimus had a more vulnerable profile with an IC50 of 6.91 μM (Figure 3E); thus, we continued using 200 nM for the subsequent experiments. Simultaneously, we defined dosage ranges on which the beating functionality was preserved as an attempt to use this heart organoid system as a preclinical setting evaluating cardiac tolerance with no electrophysiological perturbations. To that end, we subcategorized compound dosages depending on their impact on heart organoid spontaneous beating. SB202190 showed the safest profile among the three compounds, preserving average beating rates compared to untreated organoids from 100 μM downwards (Figure 3D). TAC and Sirolimus also displayed good tolerance for cardiac functionality, spotting safe dosages starting from 12 to 2.5 μM downwards, respectively (Figures 3B,F). Taken altogether, we decided to continue using concentrations of 10 μM (SB202190 and TAC) and 200 nM (Sirolimus), all dosages below the IC50 and offering a safe cardiac functionality tolerance in terms of cardiomyocyte beats.

### Inhibition of p38-MAPK in hHO can suppress COL1A1 expression upon TGF-β1 treatment

**FIGURE 4:**
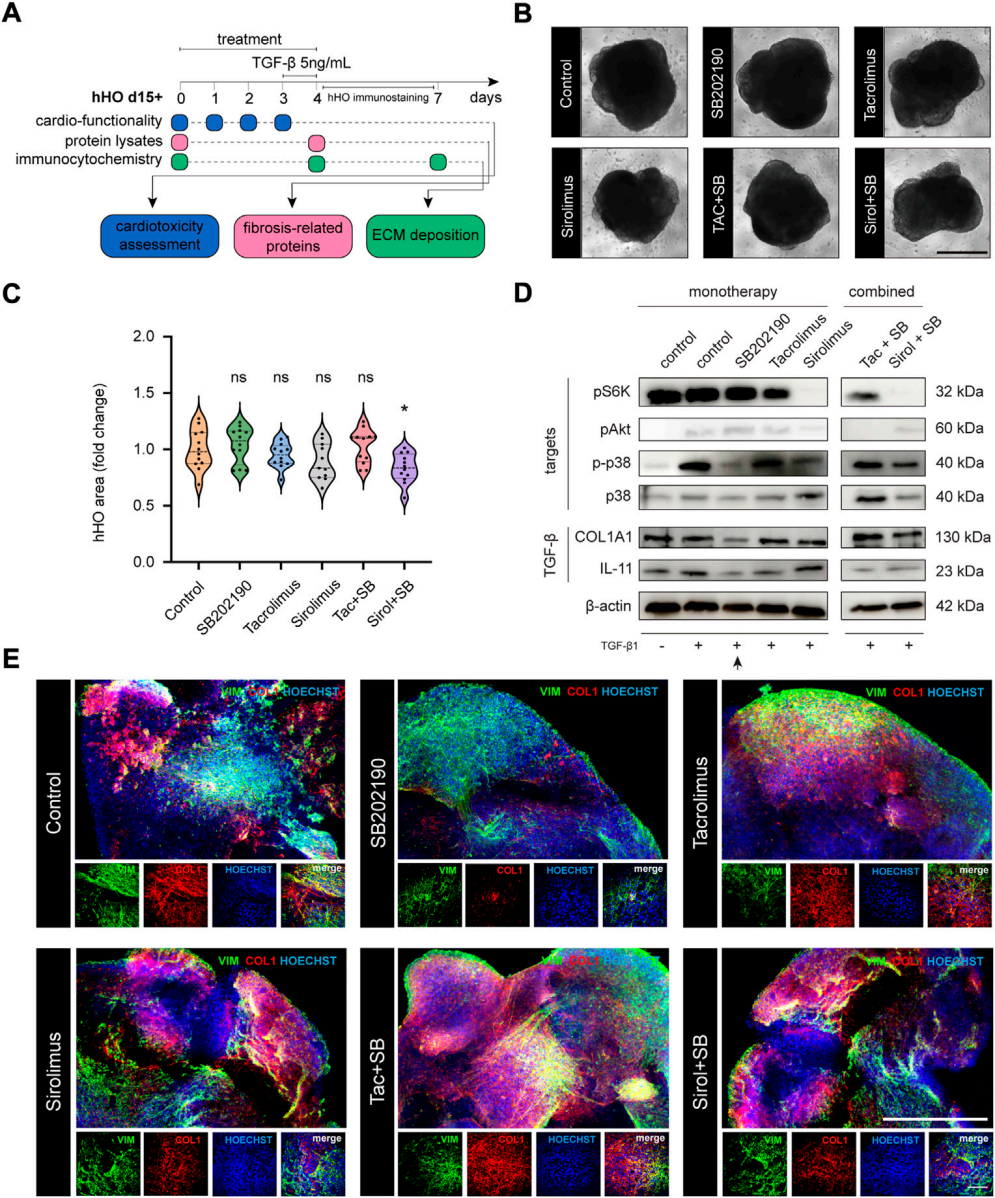
Monotherapy and combo-based chemical evaluation of antifibrotic compounds in human heart organoid modeling reactive fibrosis. **(A)** Schematic representation of the experimental workflow in hHOs. **(B)** Phase contrast microscopy for hHOs d15 + 3+1 (scale bar: 500 μm). **(C)** hHO area size after 96 h of compound treatment [relative to control [FC], *n* ≥ 11, two-tailed, unpaired *t*-test]. **(D)** Western blot analysis in hHOs for pathway targets upon different treatments and TGF-β1 stimulation [pS6K, pAkt, p-p38, p38] and TGF-β1 fibrosis-related responsive genes [COL1A1, IL-11]. **(E)** Immunocytochemistry analysis of COL1A1 expression in hHOs treated with SB202190 (10 μM) alone or in combination with Tacrolimus (10 μM) and Sirolimus (200 nM) and 5 ng/ml TGF-β1 stimulation for 24 h [scale bar: 500 μm; inset: 100 μm].

To elucidate the causal role of the RAS signaling activation in a multicellular context, we again explored the antifibrotic ability of SB202190, TAC, and Sirolimus in activated cells upon TGF-β1 stimulation in human heart organoids (Figure 4A) systematically. No treatment provided proliferative disadvantages in monotherapy, being all treated organoids in similar sizes to the untreated condition (Figures 4B,C upper panels). This phenotype ensured that results were reliable and unlikely explained by cellular loss or cell death. We verified the downstream inactivation of the signaling pathways by western blot, verifying the continued effective concentrations in heart organoids (Figures 4D,E). Next, we confirmed that the TGF-β1 stimulation triggered a fibrotic-like response in hHOs. TGF-β1 treated heart organoids significantly elevated mRNA expression levels on *IL-11* and *ACTA2* (Supplementary Figure S7A) and strongly increased protein levels of COL1A1 (Supplementary Figure S7B). Notably, whereas Sirolimus showed reduced concentrations of COL1A1 in CFs, this phenotype is lost in heart organoids, leaving SB202190 as the only compound that effectively reduced TGF-β1 driven COL1A1 expression (Figure 4E). These results indicate that the non-fibroblast populations in the heart have a profound impact on the therapeutic responses of treatments against heart diseases, being able to disrupt the beneficial effects of already described antifibrotic alternatives. Sirolimus-treated organoids showed rescued levels of IL-11 and phospho-p38, indicating that sustained and ectopic signaling of this interleukin from non-fibroblast populations could nullify its antifibrotic effects in isolated CFs through the restoration of phosphorylation levels of p38 MAPK.

### Tacrolimus and Sirolimus revert the cardiac antifibrotic properties of p38-MAPK inhibition in hHOs

Since combinational treatment of SB202190 with immunosuppressors could effectively suppress the COL1A1 expression in our primary cell line of human cardiac fibroblasts, we aimed to verify these results in the human heart organoid. Again, we confirmed that treated organoids had similar sizes to the untreated condition, except for the combination of SB202190 with Sirolimus. This treatment showed organoid decreased size (Figure 4C) with normal morphology (Figure 4D). In addition, molecular targets of each compound were validated individually upon treatment (Figure 4D). After combined treatment, we found massive ECM depositions under confocal microscopy upon pathological stimulation with TGF-β1, confirmed by western blot. The SB202190 antifibrotic effects reverted and were rescued by the concomitant treatment with the immunosuppressors TAC and Sirolimus (Figures 4D,E). Again, IL-11 levels were rescued, and a severe fibrotic reactivation was found correlative to restoration in phosphorylated levels of p38, suggesting that immunosuppressants might have deleterious effects on the antifibrotic inhibition of the p38-MAPK pathway, a non-cell-autonomous phenotype only explained by the molecular interactions in the context of the human heart.

## Discussion

Exploiting cardiac fibroblast targeting to understand how these specific cellular lineages develop and contribute to the activation of either reactive or replacement fibrosis upon cardiac injury requires detailed knowledge of the signaling that regulates fibroblast development and reactivation.

**FIGURE 5:**
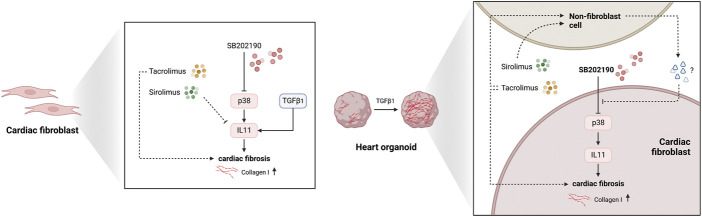
The biological model for the effects of Sirolimus and Tacrolimus effects in fibroblast and non-fibroblast populations. Illustration representing the 2D and 3D models explaining the non-cell-autonomous molecular phenotypic divergences with Sirolimus and Tacrolimus reverting the antifibrotic-response of p38-MAPK inhibition derived from SB202190.

Several pathways have been identified contributing to pathological fibroblast reactivation (Piersma et al., 2015; Ma et al., 2018; Jiang et al., 2021), a phenotype that is ultimately tissue-specific and context-dependent (Zeisberg and Kalluri, 2013; Taroni et al., 2017). Due to the embryonic heterogeneity of fibroblast lineages’, it is difficult to set the basis of common denominators driving reactive fibrosis in disease. Here we demonstrated that the RAS signaling pathway plays an imperative role in activating replacement and reactive fibrosis. This pathway has pleiotropic effects in the cellular signaling (Downward, 2003; Molina and Adjei, 2006), and exacerbated proliferative stimuli could contribute to fibroblast proliferation and activation of fibrosis pathways. Several studies attempted to target fibroblast proliferation (Travers et al., 2016; Kurose, 2021) to ameliorate large deposition of ECM, yet most of them are oversimplified studies targeting one lineage of cardiac fibroblast in isolation. Recently, several research was conducted at a single cell level resolution, aiming to identify transcriptional waves that activate fibrosis pathways upon concrete stimuli (Stone et al., 2019). Several factors, including inflammation-related pathways and cytokine activity, can finely modulate fibrotic responses (Nicoletti and Michel, 1999; Kania et al., 2009; Thomas and Grisanti, 2020). Yet, it is unknown whether the signaling driving pathological states is cell-autonomous or controlled by ectopic key factors. The human heart is a molecularly complex organ since its functionality relies on multicellular populations signaling in different directions to orchestrate a proper electrophysiological and biochemical functionality (Skelly et al., 2018; Wang et al., 2020). Our study addressed this problem by studying cardiac signaling in an iPSC-derived heart organoid model that enables the recapitulation of disease phenotypes in a developing organoid that matches with fetal cardiac tissue at the cellular, structural and transcriptomic levels (Lewis-Israeli et al., 2021). iPS cells (Takahashi and Yamanaka, 2006; Takahashi et al., 2007) have introduced revolutionary concepts in precision medicine through the potential generation of organoids that can recreate developmentally relevant models under fully defined, reproducible, and efficient conditions (Turhan et al., 2021). This study used a differentiation protocol published by Lewis-Israeli and colleagues to produce human mini hearts composed of organoid-derived cardiac myocytes (oCMs), endothelial cells (oECs), endocardial cells (oEnCs), epicardial cells (oEPI), and cardiac fibroblasts (oCFs). This model enables the creation of preclinical platforms like the one in the present study that provided an assessment system for treating cardiac fibrosis in more physiologically relevant conditions. Tacrolimus and Sirolimus are commonly used in clinical practice as immunosuppressants that follow the treatment after organ transplantation to avoid the immune rejection (Kahan et al., 1998; Plosker and Foster, 2000; Morelon et al., 2001). Beyond their immunosuppressor activity, these compounds are considered relatively safe (Sallam et al., 2022) and FDA-approved for the treatment of concrete disease conditions, including the treatment of perivascular epithelioid cell tumors for the case of Sirolimus (Wagner et al., 2010). Some studies have successfully explored the antifibrotic properties of these compounds *in vitro*, usually in isolated models of fibroblast primary cultures (Nagano et al., 2006; Tulek et al., 2011). A relevant study showed that interleukin-11 (IL-11) is intrinsically associated with the activation of the fibrotic pathways, correlative and causative of reactive fibrosis and ECM depositions (Schafer et al., 2017). In our study, we have systemically evaluated inhibitors for the downstream activators of the RAS pathway, both in monotherapy and combo treatments. In cardiac fibroblasts, p38 2i inhibitor SB202190 and Sirolimus showed effectiveness in reducing COL1A1 upon TGF-β1 stimulation when treating fibroblasts in monotherapy. The combined treatment showed a nearly complete suppression of COL1A1, an effect that was possibly associated with the attenuation of phosphorylation levels of p38. In principle, these results are thought to be promising since this study explores an effective combined treatment with a low cytotoxicity profile that ameliorates the TGF-β1-driven fibrosis. In the combined treatment, IL-11 levels are reduced, indicating that IL-11 could be associated mechanistically with the phosphorylation levels of p38. The successful suppression of COL1A1 through reduced IL-11 levels indicates that this phenotype could be sustained cell-autonomously in the cardiac fibroblast populations. However, our iPSC-derived 3D-human heart organoid model showed that this treatment efficacy is reverted and worsened when TGF-β1 treated organoids are therapeutically supplemented with SB202190 together with immunosuppressors (Figure 5). The overlapping nodes in the signaling pathways are worth investigating in future studies to highlight the importance of recreating the physiological environment and revisiting therapeutic strategies thought to be safe. Since p38, JNK, and ERK mitogen-activated protein kinases (MAPKs) are independent activators of the subsequent hierarchical kinase cascades (MAPKK, MAPKKK), chemical p38-MAPK inhibition induces the suppression of ERK downstream effectors. The contribution from Tacrolimus and Sirolimus in rescuing phosphorylation levels of p38 could be taking place at multiple levels, suggesting an effect of the immunosuppressant in non-fibroblast populations. It is broadly recognized that secretory phenotypes contribute to non-cell-autonomous effects in cardiac fibrosis after myocardial infarction. Different stimuli, including cellular senescence as part of the aging process or the cardiac remodeling after the injury itself, could ultimately contribute to abnormal ECM depositions and initiate reactive fibrotic responses in physiological settings, as suggested by different studies (Zhu et al., 2013; Hall et al., 2021).

Our human organoid platform enables multicellular crosstalk between cells in the heart, solving the bias to study cardiac fibroblast in isolation and allowing a better understanding of the mechanistic insights and cardiotoxicity-related phenotypes of these compounds. Our model will also contribute to building a 3D system that describes and interrogate the establishment of those developmental programs and mechanisms of fibrosis activation, providing inroads for ectopic manipulation to control the adverse effects of cardiac fibrosis in disease, a maladaptive process with associated morbidity and mortality. We acknowledge the limitations of this study since *in vitro* human cardiac models are complementary tools allowing mechanistic interrogation in an over-reductionist way (Thomas et al., 2022). Among these complementary tools, a finely-tuned integration of 3D-biomaterials-based methods, 3D-bioprinting, Human Engineered heart tissues (EHTs/muscle), micro-tissues, and MPSs sensors in combination with vascularized heart organoids and 2D cultures based on hiPSC-derived cardiomyocytes differentiation will provide insightful progress in the field of precision medicine and cardiac disease modeling (Cho et al., 2022). This study nonetheless reports a reliable preclinical model for evaluating drug cardiotoxicity and assessing cardiac fibrosis phenotypes in 3D human heart organoids derived from human iPS cells, endorsing their preclinical value and exploring imperative roles of the heart microenvironment in response to treatments, opening new questions and highlighting the importance of the development of precision medicine in cardiovascular research.

## Data Availability

The datasets presented in this study can be found in online repositories. The names of the repository/repositories and accession number(s) can be found below: Retrospective bioinformatical analysis performed in this study was performed using R2: Genomics analysis and visualization platform (http://r2.amc.nl/). The single cell RNA-Seq used for analysis is available under the accession code GSE133452, GSE132146 and GSE97458.
