# Supplementary material for: Immunosuppressants Tacrolimus and Sirolimus revert the cardiac antifibrotic properties of p38-MAPK inhibition in 3D-multicellular human iPSC-heart organoids
Source: Front Cell Dev Biol. 2022 Nov 11;10:1001453. doi: 10.3389/fcell.2022.1001453 (PMC9692097; doi:10.3389/fcell.2022.1001453)
Supplement: Supplementary file 1 [file DataSheet1.DOCX]

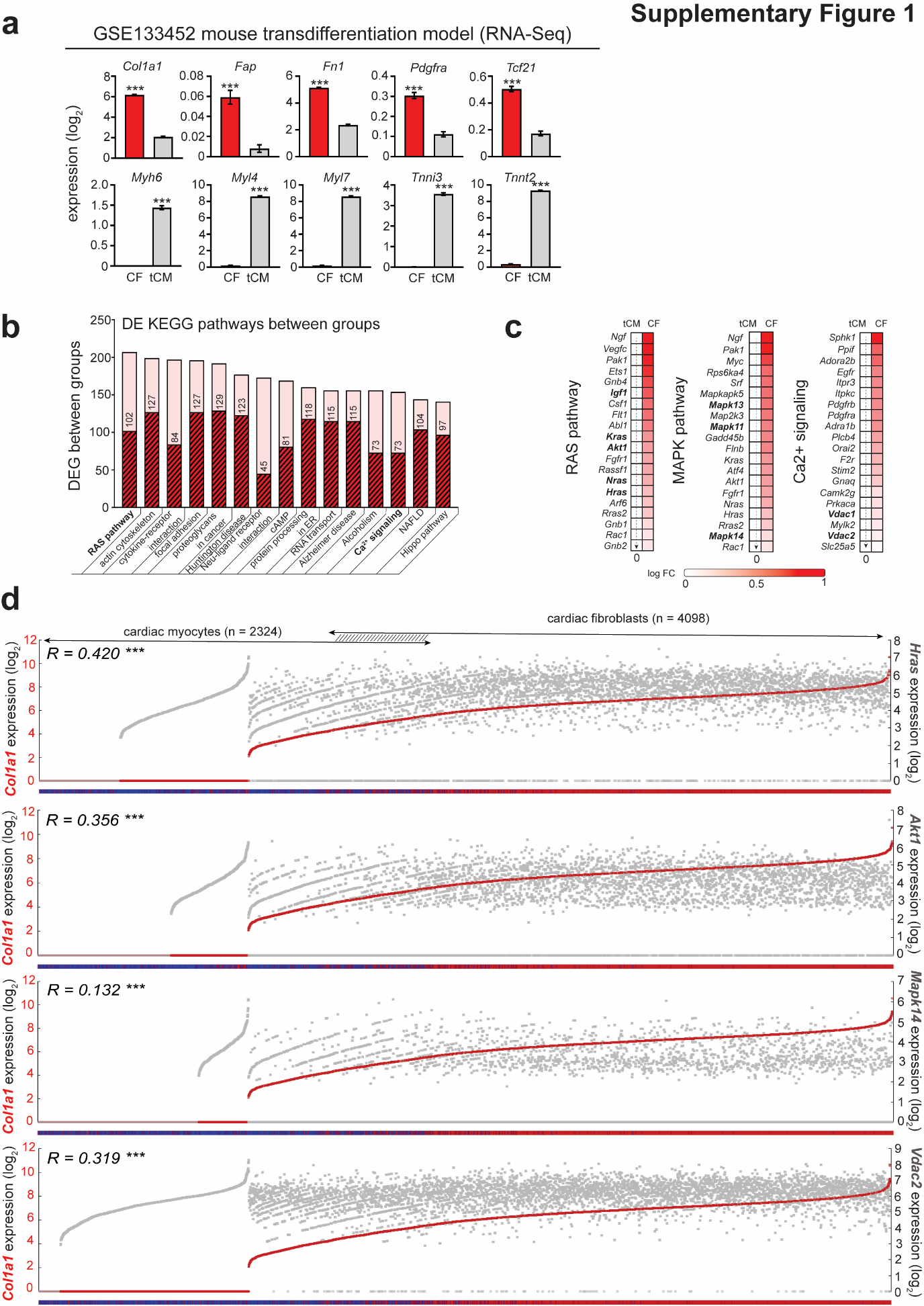


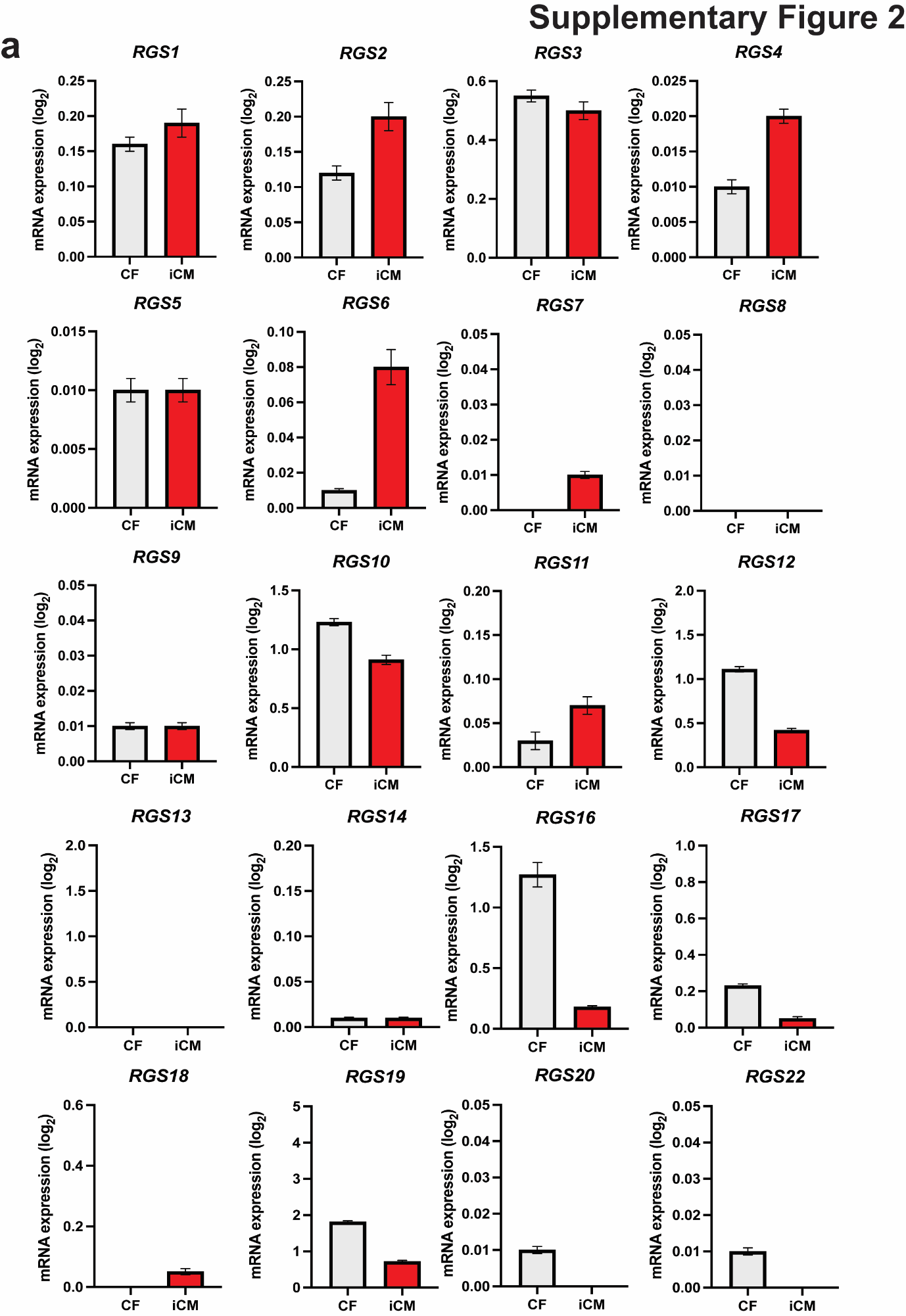


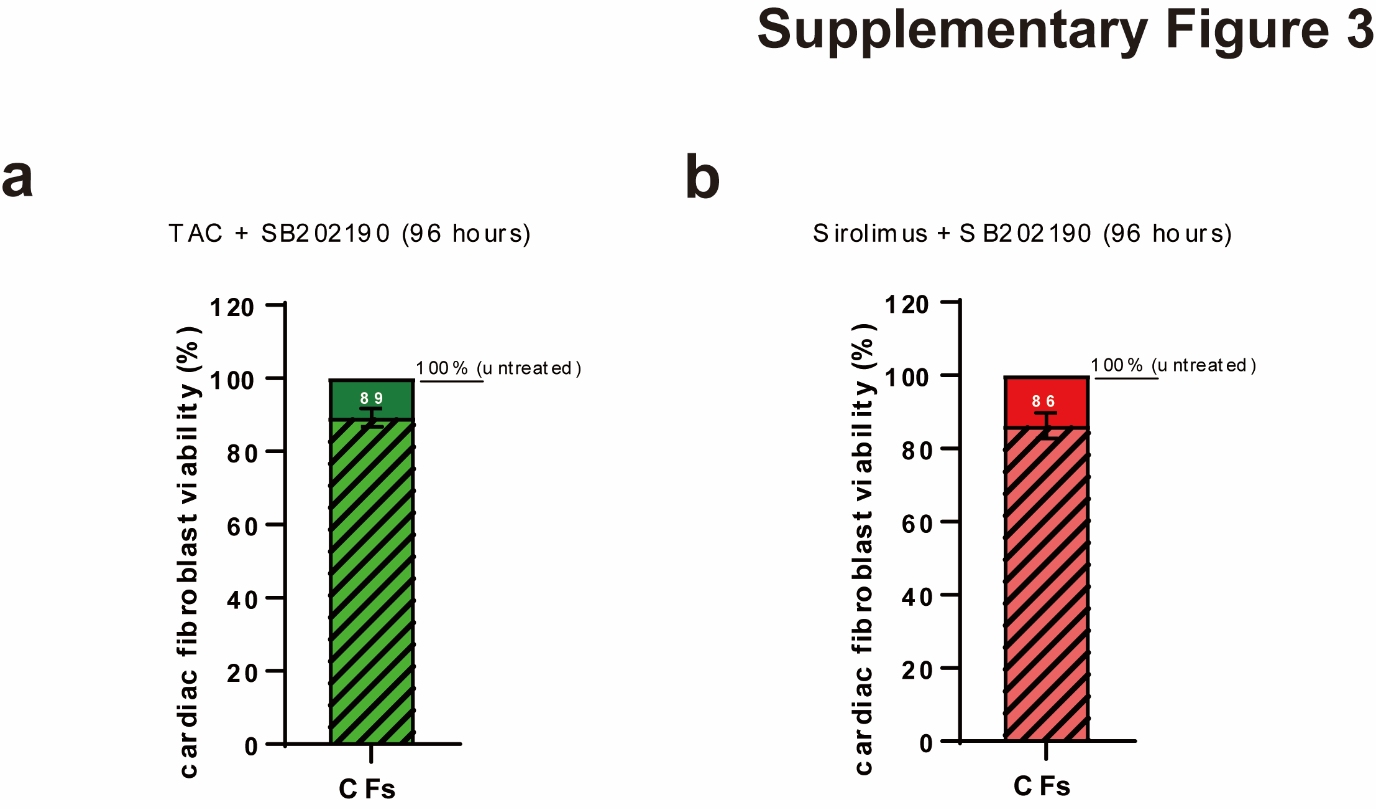


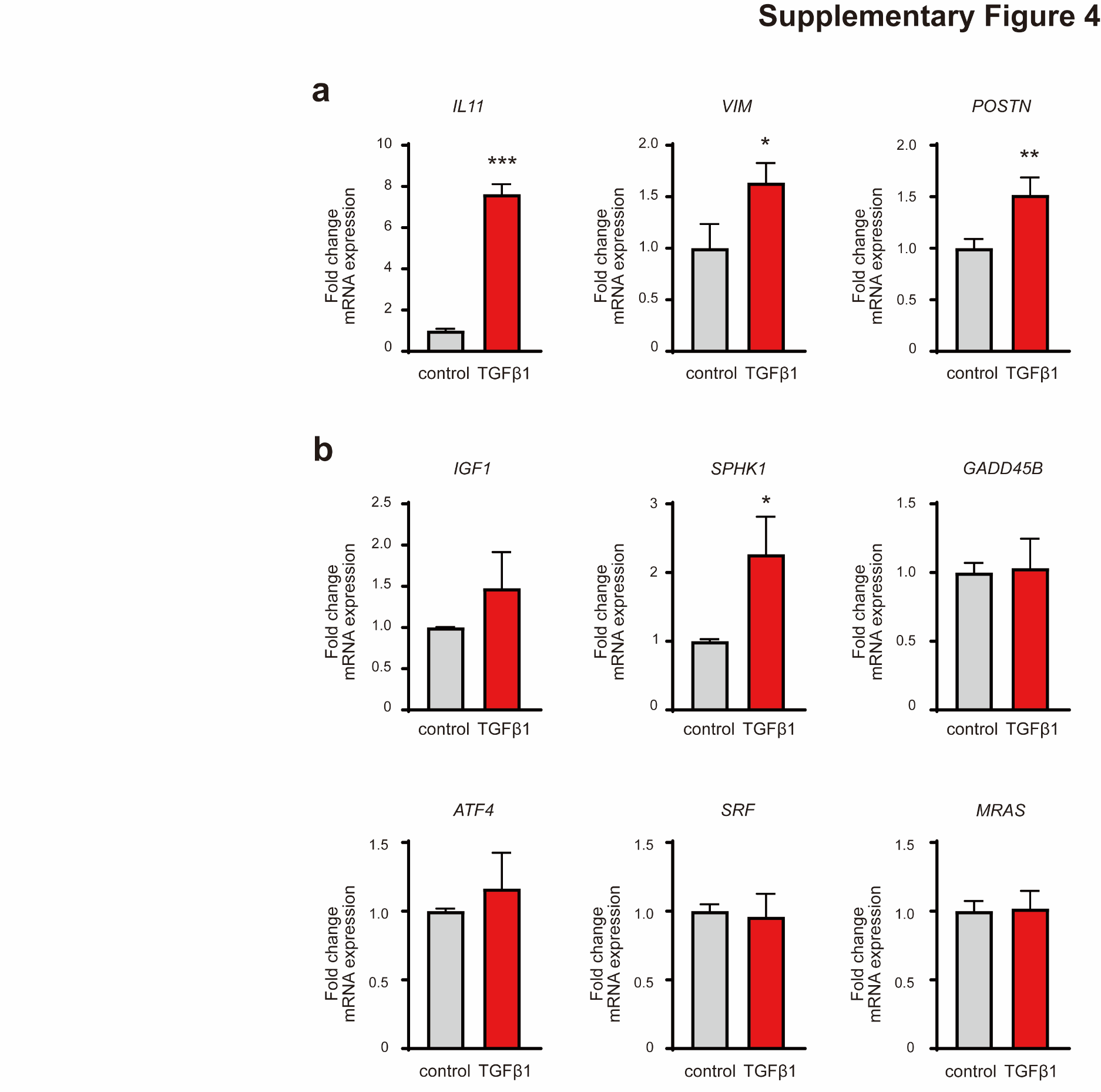


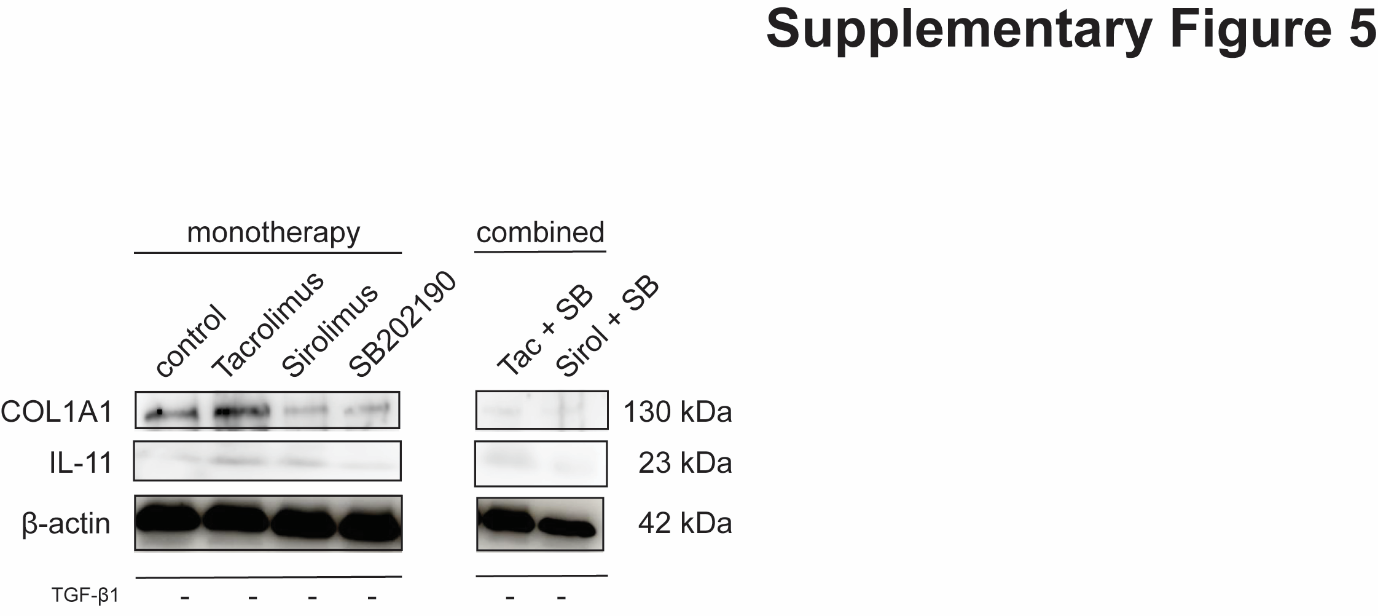


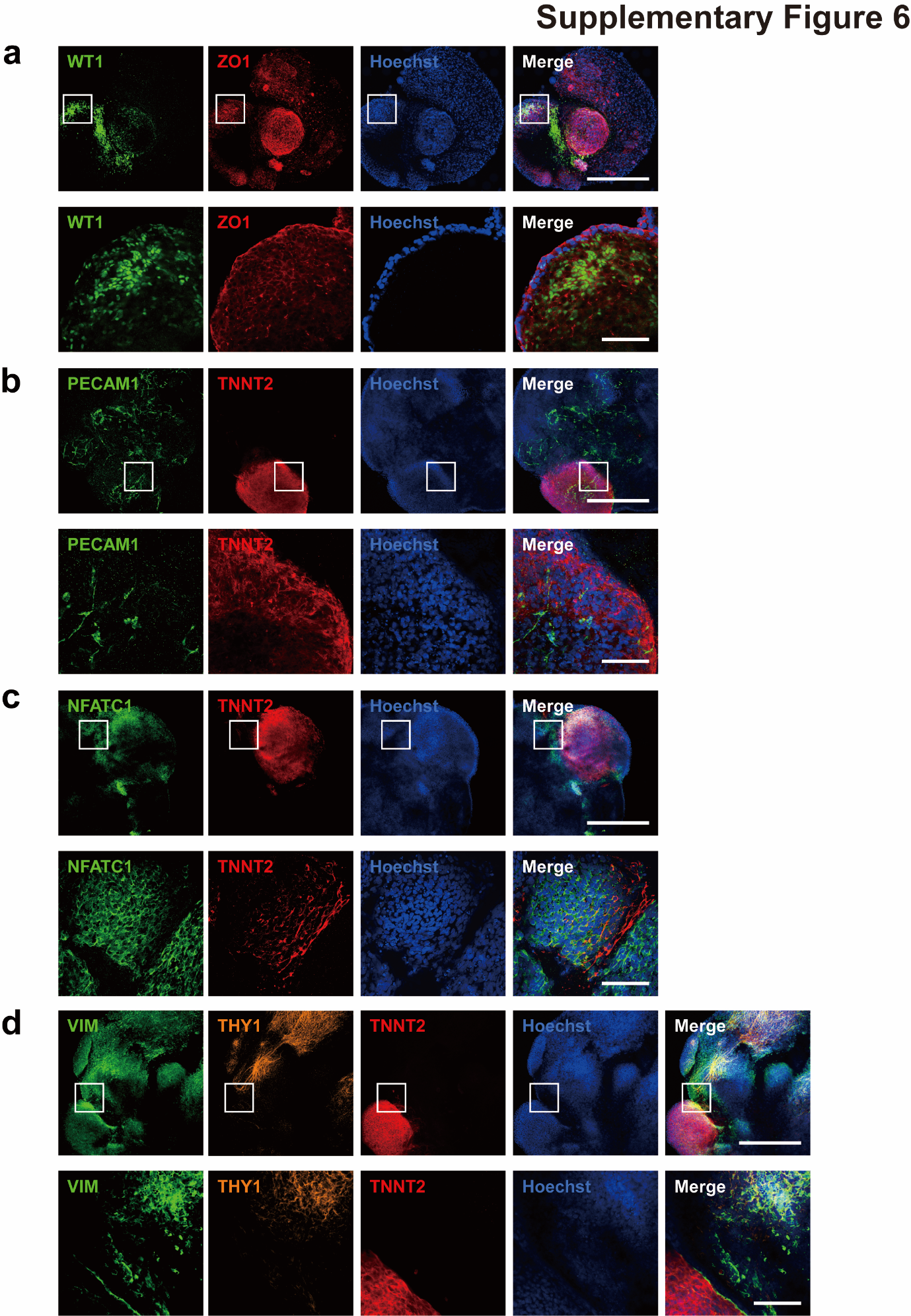


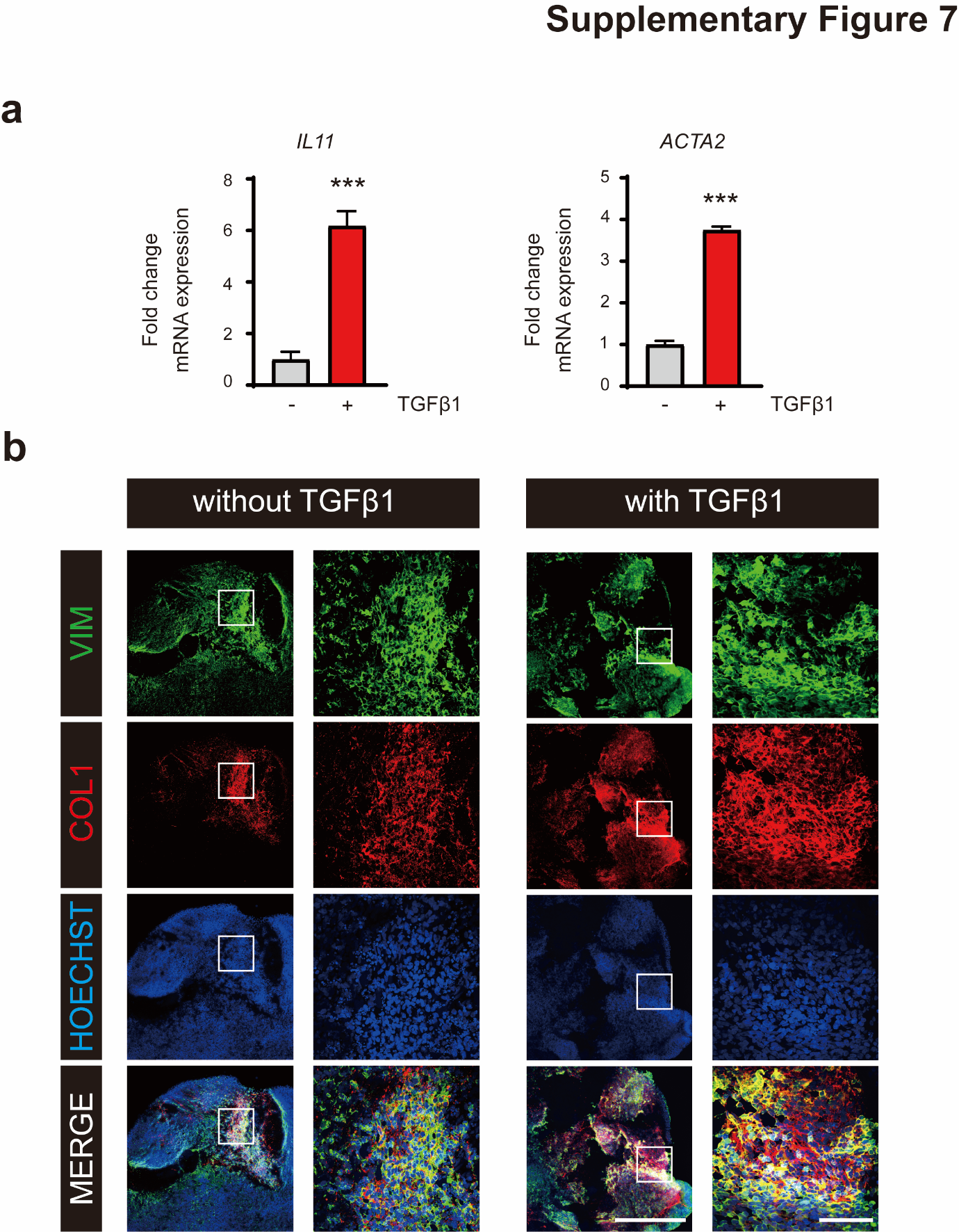


Supplementary Table 1. Primers for qPCR

| Gene | Forward primer (5’ to 3’) | Reverse primer (5’ to 3’) |
| --- | --- | --- |
| IL11 | CGAGCGGACCTACTGTCCTA | GCCCAGTCAAGTGTCAGGTG |
| VIM | GTTTCCAAGCCTGACCTCAC | GCTTCAACGGCAAAGTTCTC |
| POSTN | TGTTGCCCTGGTTATATGAG | ACTCGGTGCAAAGTAAGTGA |
| IGF1 | GCTCTTCAGTTCGTGTGTGGA | GCCTCCTTAGATCACAGCTCC |
| SPHK1 | GGCTGCTGTCACCCATGAA | TCACTCTCTAGGTCCACATCAG |
| GADD45B | TACGAGTCGGCCAAGTTGATG | GGATGAGCGTGAAGTGGATTT |
| ATF4 | ATGACCGAAATGAGCTTCCTG | GCTGGAGAACCCATGAGGT |
| SRF | CGAGATGGAGATCGGTATGGT | GGGTCTTCTTACCCGGCTTG |
| MRAS | ACAAGGTCGATTTGATGCACT | GCACTGGTTTCTATGTACGGAAT |
| ACTA2 | TCAATGTCCCAGCCATGTAT | CAGCACGATGCCAGTTGT |
| GAPDH | TGATGACATCAAGAAGGTGGTGAAG | TCCTTGGAGGCCATGTGGGCCAT |

**SUPPLEMENTARY FIGURE LEGENDS**

**Supplementary Figure 1. Single-cell transcriptomics driving to activation of Col1a1 in a transdifferentiation model of mouse cardiac fibroblasts**

**a** Expression analysis for lineage identification of the single cell dataset GSE133452 showing transcriptional expression of cardiac fibroblast-related genes (top) and cardiomyocytes-related genes (bottom) **b,** KEGG analysis for the identification of differentially expressed pathways between groups [sorted by the total number of genes in the pathway] **c,** heatmaps of representative genes overexpressed of RAS, MAPK and Ca^2+^ pathways [values represented as log FC relative to tCM (0) expression] **d,** single cell correlations between groups to HRas, Akt1, Mapk14 and Vdac2 [samples were classified individually by a crescent Col1a1 gene expression level]

**Supplementary Figure 2. GSE133452 analysis of RGS genes**

**a,** Retrospective analysis of GSE133452 of RGS genes (*, P< 0.05; **, P < 0.01; ***, P < 0.001 compared to iCMs)

**Supplementary Figure 3. Cardiac fibroblast viability to combined treatment of Tacrolimus and Sirolimus with SB202190.**

**a,** Cellular viability to 96 hours of combo treatment of TAC+SB202190 (10 μM + 10 μM; left) and Sirolimus + SB202190 (200 nM + 10 μM; right)

**Supplementary Figure 4. Cellular characterization of iPSC-derived human heart organoid composition**

**a,** mRNA expression levels (fold change) in NHCF-V cell line for evaluation of TGF-β1 responsive genes [*IL-11*, *VIM*, *POSTN*] and **b,** RAS signaling related genes (*, P< 0.05; **, P < 0.01; ***, P < 0.001 compared to control)

**Supplementary Figure 5. Cellular characterization of COL1A1 expression in response to immunosuppressants in unstimulated cardiac fibroblasts**

**a,** Western blot analysis in NHCF-V cell line for evaluation of cardiac fibrosis and TGF-β1 responsive genes [COL1A1, IL-11] in monotherapy and combo-treatment

**Supplementary Figure 6. Cellular characterization of iPSC-derived human heart organoid composition**

**a,** Immunocytochemistry analysis of WT1 and ZO-1 expression marking epicardial cells in hHOs **b,** Immunocytochemistry analysis of PECAM1 (CD31) marking endothelial cells and TNNT2 marking cardiomyocytes in hHOs **c,** Immunocytochemistry analysis of NFATC1 marking endocardial cells and TNNT2 marking cardiomyocytes in hHOs **d,** Immunocytochemistry analysis of VIM and THY1 marking cardiac fibroblasts in hHOs [scale bar: 500 μm; inset: 100 μm]

**Supplementary Figure 7. Characterization of the pro-fibrotic response to TGF-β1 in iPSC-derived human heart organoid**

**a,** mRNA expression levels of fibrosis-related genes *IL-11* and *ACTA2* (*, P< 0.05; **, P < 0.01; ***, P < 0.001 compared to unstimulated condition) **b,** Immunocytochemistry analysis of VIM and COL1A1 for the evaluation of TGFβ1-driven fibrosis in unstimulated (left) and stimulated (right) hHOs [scale bar: 500 μm; inset: 100 μm]
